# Paclitaxel and its semi-synthetic derivatives: comprehensive insights into chemical structure, mechanisms of action, and anticancer properties

**DOI:** 10.1186/s40001-024-01657-2

**Published:** 2024-01-30

**Authors:** Priyanka Sati, Eshita Sharma, Praveen Dhyani, Dharam Chand Attri, Rohit Rana, Lashyn Kiyekbayeva, Dietrich Büsselberg, Samson Mathews Samuel, Javad Sharifi-Rad

**Affiliations:** 1 Department of Biotechnology, Kumaun University, Bhimtal, Uttarakhand India; 2 Department of Molecular Biology and Biochemistry, Guru Nanak Dev University, Amritsar, Punjab India; 3 Institute for Integrated Natural Sciences, University of Koblenz, Koblenz, Germany; 4 Department of Botany, Central University of Jammu, Rahya-Suchani (Bagla), Jammu and Kashmir India; 5 Department of Biology, Brandeis University, Waltham, MA USA; 6 Department of Pharmaceutical Technology, Pharmaceutical School, Asfendiyarov Kazakh National Medical University, Almaty, Kazakhstan; 7 Department of Physiology and Biophysics, Weill Cornell Medicine-Qatar, Education City, Qatar Foundation, P.O. Box 24144, Doha, Qatar; 8 Facultad de Medicina, Universidad del Azuay, Cuenca, Ecuador

**Keywords:** Paclitaxel, Anticancer, Molecular mechanisms, Bioactive compounds

## Abstract

Cancer is a disease that can cause abnormal cell growth and can spread throughout the body. It is among the most significant causes of death worldwide, resulting in approx. 10 million deaths annually. Many synthetic anticancer drugs are available, but they often come with side effects and can interact negatively with other medications. Additionally, many chemotherapy drugs used for cancer treatment can develop resistance and harm normal cells, leading to dose-limiting side effects. As a result, finding effective cancer treatments and developing new drugs remains a significant challenge. However, plants are a potent source of natural products with the potential for cancer treatment. These biologically active compounds may be the basis for enhanced or less toxic derivatives. Herbal medicines/phytomedicines, or plant-based drugs, are becoming more popular in treating complicated diseases like cancer due to their effectiveness and are a particularly attractive option due to their affordability, availability, and lack of serious side effects. They have broad applicability and therapeutic efficacy, which has spurred scientific research into their potential as anticancer agents. This review focuses on Paclitaxel (PTX), a plant-based drug derived from Taxus sp., and its ability to treat specific tumors. PTX and its derivatives are effective against various cancer cell lines. Researchers can use this detailed information to develop effective and affordable treatments for cancer.

## Introduction

Cancer is a severe public health issue, with around six million new cases yearly. Research has identified several significant causes, including exposure to certain chemicals and types of electromagnetic radiation in the diet, environment, or workplace [1]. The level of risk associated with these exposures is a topic of much debate, prompting preventative efforts such as the US national "Smoke Out" program, which aims to limit exposure to carcinogenic chemicals [2]. Many advancements have been made in developing anticancer medications due to studies on the molecular pathways involved in cancer growth. However, despite these efforts, using chemically made medications has not significantly improved overall survival rates. Treating cancer remains a challenging task with limited success. Available treatment choices comprise surgery, radiation therapy, and systemic chemotherapy. In the chemotherapy drug category, medications like methotrexate (antimetabolites), cisplatin and doxorubicin (DNA-interactive drugs), taxanes (anti-tubulin) are most widely used in addition to other hormones, and molecular targeting drugs [3]. Chemotherapy has several disadvantages, such as cancer recurrence, drug resistance, and harmful effects on healthy tissues, which can hinder the effectiveness of anticancer drugs and negatively affect a patient's well-being. Researchers are always searching for new, improved therapies with fewer side effects to overcome these obstacles and maintain the quality of life for those living with cancer [4].

Medicinal plants have numerous advantages over artificial products, as they are non-toxic to normal human cells. Conventionally, cancer is treated with radiotherapy and chemotherapy, but unfortunately, these methods have adverse side effects that can seriously harm a patient's health. These side effects include neurological, cardiac, renal, and pulmonary toxicity. Therefore, developing an alternative strategy that utilizes anticancer medications that are more effective and less hazardous than those currently available is crucial. The National Cancer Institute, Maryland, USA has examined approximately 35,000 plant species to determine their potential anti-cancer properties. As a result, they have discovered 3000 species having repeatable anticancer efficacy [5]. To develop additional medications for treating this illness, it is indispensable to research the primary anticancer agents that have arisen from natural sources. Medicinal plants contain secondary metabolites such as flavonoids, flavones, anthocyanins, lignans, coumarins, and catechins. These bioactive molecules are responsible for the high levels of antioxidants in medicinal plants [6, 7]. Research on herbal treatments has shifted nowadays due to expensive synthetic drugs and their side effects. Significantly, interest in preventing, eradicating, and treating diseases like cancer and metabolic disorders has risen because of more deaths worldwide [8, 9]. These studies have shown that Taxus sp. and its components, especially Paclitaxel (PTX), have various biological actions.

**Fig. 1:**
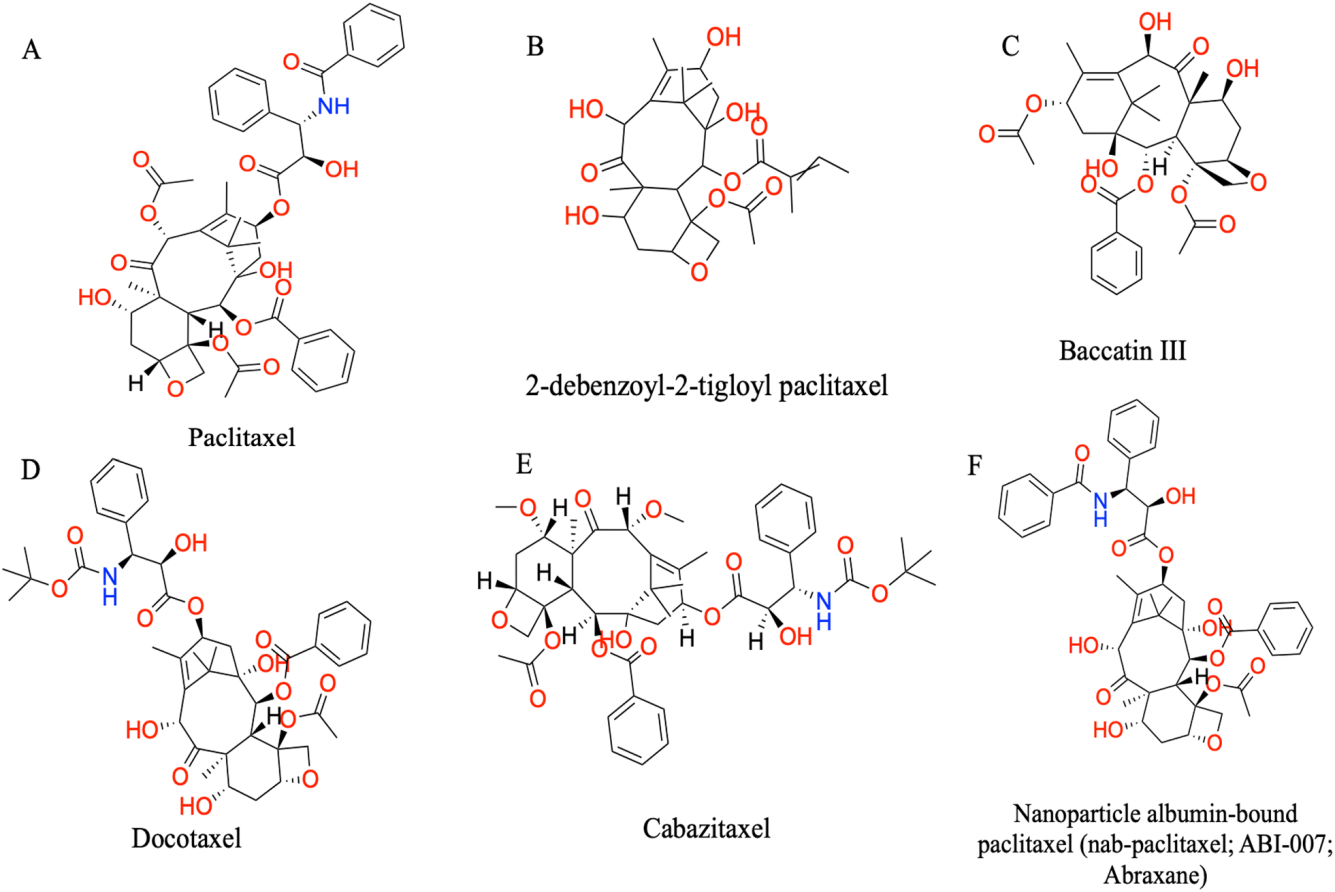
Chemical structure of paclitaxel and its derivatives. **A** Paclitaxel, **B** 2-debenzoyl-2-trigloyl paclitaxel, **C** baccatin III, **D** docetaxel, **E** cabazitaxel, and **F** Abraxane

There are at least 10 diverse species of Taxus, such as *T*. *baccata*, *T*. *cuspidata*, *T*. *wallichiana*, and *T*. *xmedia* cv. Hicksii, which contain taxol [10–12]. The first instance of Taxol, specifically *Taxus brevifolia*, was created by Wani and colleagues by utilizing Nutt (Taxaceae) and a synthetic form of the plant [13]. After the finding of Taxol, more than 400 naturally occurring taxol analogs were discovered, leading to the isolation of numerous other taxoids. Typically, Taxus species contain a relatively small amount of taxol, ranging from 0.001 to 0.06% of the dried bark's weight [14]. Taxol is most prevalent in *T. brevifolia*: bark, needles, roots, branches, seeds, and wood. PTX was first extracted from the stem bark of the western yew, *Taxus brevifolia*, in 1960s; Wani and colleagues discovered its structure in 1971. Taxol^®^ is a drug currently marketed as PTX. It was approved for clinical use in 1994, with the first medicinal application being for the treatment of ovarian cancer [15]. In 1971, researchers discovered that PTX was the active ingredient in the extract. This drug belongs to the taxane family and has a unique chemical structure (Fig. 1a) with the molecular formula C_47_H_51_NO_14_. *Taxus brevifolia*, a type of Pacific yew, was originally used to obtain this compound. However many of the species of fungus Pestalotiopsis viz, *Pestalotiopsis versicolor*, *Pestalotiopsis neglecta* (isolated from Japanese Yew tree, *Taxus cuspidata*), *Pestalotiopsis pauciseta* (isolated from *Cardiospermum helicacabum*) and *Pestalotiopsis terminaliae* was extracted from the fresh healthy leaves of *Terminalia arjuna* (arjun tree) and then examined for the ability to produce the anticancer medication taxol in a synthetic culture medium (Gangadevi [16–18]. According to [19], PTX is a vital anticancer medication to treat ovarian, breast, and lung cancer.

To treat a single patient, approximately 2 g of PTX is requisite, and this amount can be extracted from the 3–10 tree’s bark. On average, taxol has a yield of 0.015% approximately. It takes 2000–2500 yew trees to produce just one kilogram of taxol [20–22]. The tree's slow growth would cause its natural stands to be depleted entirely if it were harvested for taxol. Many researchers have used the bark of Taxus spp. instead of needles to obtain docetaxel and Taxotere, rather than PTX, to protect this crucial population [23–26]. Research on taxane diterpenoids, commonly known as taxoids, has increased over the past 20 years from a small field of natural product chemistry into a nearly $1 billion business [27]. This is because the yew develops slowly, and losing its bark kills the tree. Baccatin III or 10-deacetyl baccatin III can be made widely available as a precursor without adversely hurting the trees. The production of taxol and the recently released taxotere (docetaxel) began with 10-deacetyl baccatin III. As a result, taxol's generic name was decided upon as PTX [28]. Six unique taxane diterpenoids have been found in the seeds of *Taxus yunnanesis* and *Taxus chinensis* var. *mairei* [29].

PTX and its derivative docetaxel prevent cell mitosis by stabilizing the microtubule polymer, which leads to cell death [30, 31]. Due to a unique method of action targeting microtubule assembly, the FDA approved PTX for managing ovarian and breast cancer [32, 33]. Currently, PTX is used to treat breast, ovarian, and non-small cell lung cancer by itself or in combination with other medications [34, 35]. It operates by obstructing microtubules' typical cell-division activity. PTX has anticancer properties, as the National Cancer Institute shows, that examined plant extracts from thousands of different species. PTX slows the advancement of the cell cycle, mitosis, and the proliferation of cancer cells by promoting tubulin assembly into microtubules and inhibiting their dissociation [36]. Contrary to previous tubulin-binding anticancer drugs, this one allows tubulin to assemble into microtubules [37, 38]. Clinical trials are being carried out for degenerative brain diseases, and it is employed in coronary heart disease, skin conditions, renal and hepatic fibrosis, inflammation, and axon regeneration [39, 40]. Beyond oncology, paclitaxel has found important applications in the field of vascular devices such as stents and balloons. The paclitaxel-coated devices are used to address the restenosis in blood vessels following procedures like angioplasty (https://www.fda.gov/medical-devices/cardiovascular-devices/paclitaxel-coated-balloons-and-stents-peripheral-arterial-disease. This strategy improves the effectiveness of treatments and subsequently improves patient outcomes, marking a substantial advancement in the fields of vascular medicine and interventional cardiology.

The fact that PTX is widely disseminated throughout the body volume demonstrates its affinity for the bound albumin protein. The liver mainly carries out PTX's metabolism and involves biliary excretion. When PTX is taken in its entirety, 6–10% of it is found in urine as an unaltered medication, and 70% of it is excreted in feces with the metabolite 6-hydroxy PTX [41]. Despite PTX's impressive anticancer activity, its lesser solubility in water and other solvents restricts its use and bioavailability. However, PTX was blended with  cremophor and dehydrated alcohol at a 50:50 v/v ratio to overcome the solubility problem, but this alteration revealed significant adverse consequences, including hypersensitivity and incompatibility with standard intravenous infusion settings [42]. P-glycoprotein (P-gp), a substance that PTX is a substrate of, expels the drug from cells, leading to the advance of drug resistance [43]. Many P-gp inhibitors were co-administered with PTX to overcome solubility issues, including verapamil [44] and valspodar [45]. However, the outcomes were unsatisfactory due to these inhibitors' toxic effects and ability to alter the pharmacokinetics and biodistribution of PTX.

In addition, this review tries to elucidate the various forms of paclitaxel, their chemical makeup, semi-synthetic derivatives, and how these substances work as anticancer medications. Scientific data supporting their classification as anticancer compounds and their current and historical uses as beneficial remedies are also discussed. The review will contribute to a better understanding of plant-based cancer therapies, including different PTX and its derivatives.

## Review methodology

In this review, we analyzed and discussed recent data on PTX's chemo-preventive and chemotherapeutic effects. The most recent studies involving cellular and molecular anticancer mechanisms were reviewed based on pharmacological evidence in specialized databases such as PubMed/MedLine, SCOPUS, Google Scholar, and the TRIP database. A database study revealed that from 1965 to 2024, approximately 400 papers have been published on various aspects of paclitaxel. For the search, we used the following MeSH terms: “paclitaxel/chemistry”, “Taxol”, “Anticancer Chemotherapeutic Agents”, Anticancer therapy use”, “Tumour immunotherapy”,” Taxol/paclitaxel kills cancer cell”, Carcinoma cell line/apoptosis. We included preclinical studies highlighting the mechanisms of action, signaling pathways, and molecular mechanisms of action of andrographolide. The studies that did not have precise pharmacological mechanistic results explained or that used homeopathic remedies were excluded. Plant taxonomy was validated with World Flora Online, and chemical formulations with PubChem [46, 47].

## General characterization of PTX and its semi-synthesis derivatives

One of the most important secondary metabolites known to have anticancer properties is taxol, a complex diterpene derived from Taxus spp. with a molecular weight of 853.9 Da. Its chemical name is 5, 20-epoxy-1,2,4,7,13-hexahydroxytax-11-en-9-one-4, 10-diacetate-2-benzoate 13 esters with (2R,3S)-N-benzoyl-3-phenylisoserine [48]. Taxol's structural foundation comprises the A, B, and C ring systems, each containing various functional groups such as two hydroxyl groups, one benzoyl group, two acetyl groups, and an oxetane ring. The C13 side chain, specifically (2′R,3′S)-N-benzoyl-3′-phenylisoserine, also contains hydroxyl and benzoyl functional groups and is connected to the core at C13. Although the ester group at the C2 position has been altered, 2-debenzoyl-2-tigloyl PTX (Fig. 1b), the initial natural derivative of PTX, still displays tubulin binding activity [49]. The PTX molecule is made up of an amide tail and a tetracyclic core known as baccatin III (Fig. 1c). The core rings are referred to as rings A (a cyclohexene), B (a cyclooctane), C (a cyclohexane), and D (an oxetane) in that order.

A derivative of taxane, called PTX-TTHA (PTX-triethylenetetramine hexaacetic acid conjugate), was created using PTX, triethylenetetramine hexaacetic acid, dimethylaminopyridine, and triethylamine. This semi-synthetic, water-soluble derivative showed improved PTX cosolvent toxicity, better water solubility, and efficacy against triple-negative breast cancer. PTX-TTHA caused cell apoptosis, decreased cell proliferation, and mediated TUNEL-positive apoptotic cells [51]. Additionally, docetaxel (Taxotere^®^), a semi-synthetic alternative to PTX, was found to be highly practical and, in some cases, more effective than PTX. The FDA has approved Docetaxel as a medication (Fig. 1d) for head and neck cancer, advanced breast cancer, and metastatic hormone-refractory prostate cancer (HRPC) treatment. Despite being significant drugs for treating various cancers, PTX and docetaxel are less effective due to drug resistance. Both drugs are vulnerable to multidrug resistance.

An increased expression in taxanes' resistance is done by the multidrug resistance gene, which encodes the P-glycoprotein gene, mostly related to increased expression. Cabazitaxel (Fig. 1e) is superior to PTX and docetaxel owing to methoxy groups' presence at C7 and C10, which results in a lower affinity for P-gp. Due to its enhanced properties, this drug effectively treats tumors resistant to docetaxel [52]. If a patient with metastatic HRPC has previously used docetaxel–prednisone therapy, it is recommended to use prednisone and cabazitaxel for treatment [52]. The clinical advantages of cabazitaxel's unique capacity to cross the blood–brain barrier (BBB) have not yet been studied [53]. Cabazitaxel significantly improved the cytotoxicity in docetaxel-sensitive cell lines, including lymphoblastic leukemia, promyelocytic leukemia (HL60), cervical adenocarcinoma (KB), and breast cancer (Calc18). The drug also proved effective in cancer cell lines that had previously been resistant to docetaxel, such as document number 1/DOX, document number 1/TXT, document number 1/VCR, HL60/TAX, Calc18/TXT, and KBV1 [54]. The resistance factor ratios of docetaxel were between 4.8 and 5.9, while those of capaztaxel ranged from 1.8 to 10.

A nanoparticle albumin-bound PTX, Abraxane (Fig. 1f), is a unique PTX version that does not contain CrEL. The particles of PTX are stabilized by human albumin 130 nm in size, making it safe for intravenous administration without the possibility of capillary occlusion [55]. To prepare Abraxane, it can be mixed with normal saline in doses ranging between 2 and 10 mg/mL. This differs from CrEL-PTX, resulting in a smaller infusion volume and duration [56, 57]. Additionally, unlike CrEL-PTX [58], Abraxane does not run the hazard of plasticizer leakage from infusion bags and can, therefore, be produced in standard plastic IV infusion bags.

**Fig. 2:**
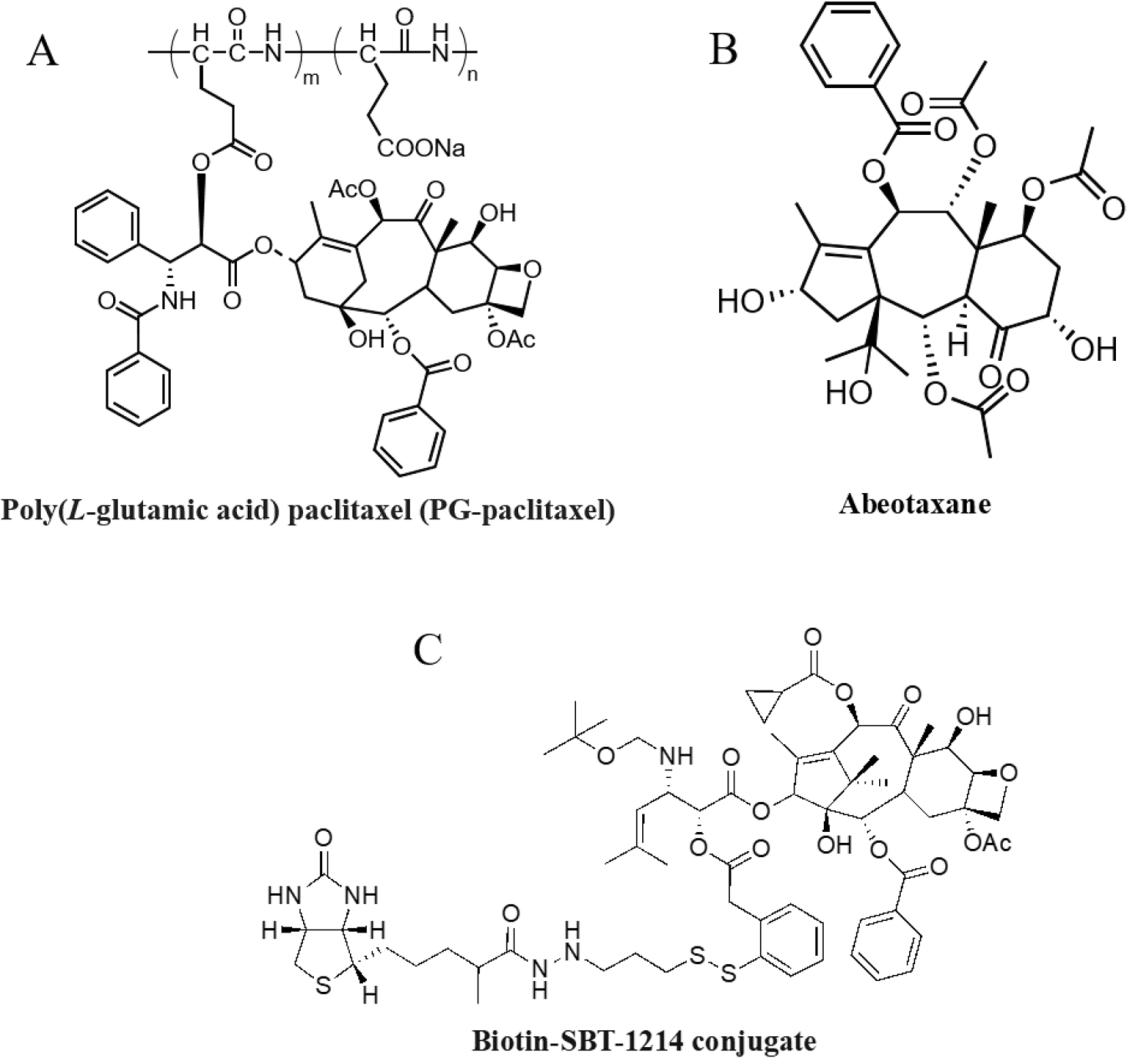
Chemical structure of poly(l-glutamic acid) paclitaxel (PG-paclitaxel) (**A**); conjugates abeotaxane (**B**); biotin-taxoid (SB-T-1214) (**C**)

PTX can be conjugated with biodegradable polymer Poly(l-glutamic acid) a water-soluble, having carboxylic acid side chains (Fig. 2a). The resulting conjugate is extremely water-soluble (> 20 mg/kg) and does not require CrEL for formulation. In chemotherapy-naive patients with advanced NSCLC, PG-PTX revealed equal effectiveness with less myelotoxicity but higher neurotoxicity than gemcitabine or vinorelbine [59]. When used as second-line therapy for NSCLC, PG-PTX exhibited survival rates that were comparable to those of docetaxel while having higher rates of neurotoxicity and lower incidences of alopecia, neutropenia, and febrile neutropenia. Utilizing the identical isoserine C13-side chain as SB-T-1214, a library of 7, 10-modified PTX, cabazitaxel, and ortataxel analogs was published [60], several of these taxanes showed outstanding to good efficacy against various cancer cell lines. Numerous "abeo-taxanes" (Fig. 2b) have been produced by altering the C7- and C9-hydroxyl groups. These taxane skeletons are derived from baccatin III via skeletal rearrangement. These abeo-taxanes showed good potency when applied to cancer cell lines resistant to PTX, vinblastine, and doxorubicin [61].

A self-immolating disulfide linker was used to create biotin-taxoid (SB-T-1214) conjugates (Fig. 2c), including single-walled carbon nanotube (SWNT) nano-conjugates [62, 63]. These conjugates demonstrated remarkable efficiency with far lower toxicity to normal human cells and very good internalization of cancer cells via receptor-mediated endocytosis.

**Fig. 3:**
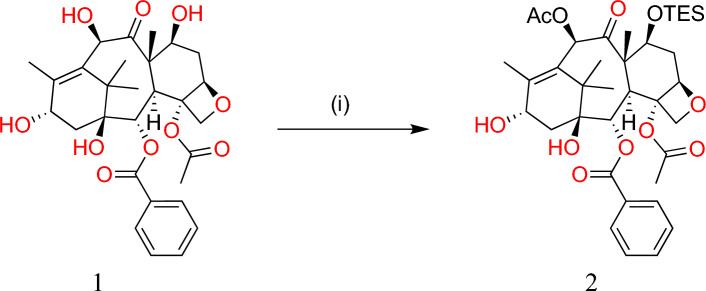
Synthesis of paclitaxel derivative (**2**) (reaction condition i) TESCl, pyridine, room temp., 5 min; (reaction condition 2) LiHMDS, CH_3_COCl, THF, − 40 °C, 30 min

**Fig. 4:**
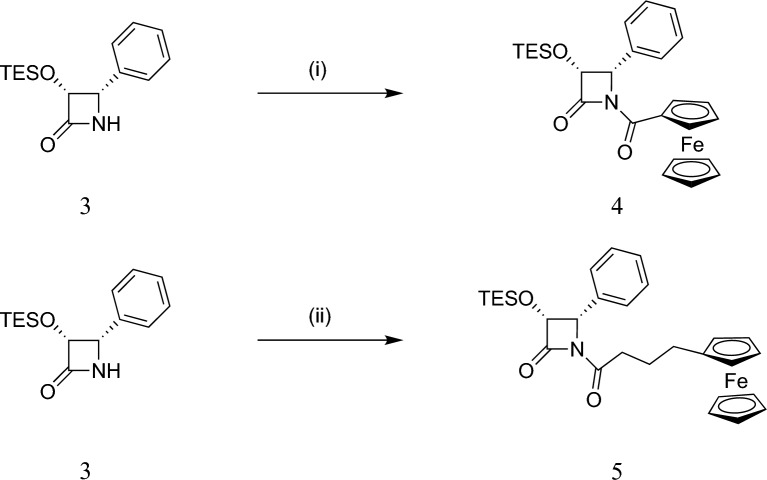
Synthesis of *N*-ferrocenyl-substituted azetidin-2-ones (**4**) and (**5**). Reaction conditions: (i) FcCOCl (Fc = ferrocenyl), Et_3_N, DMAP, DCM, room temperature, 2 h; (ii) Fc(CH_2_)_3_COOH, DIC, DMAP, DCM, room temperature, 24 h

Many PTX and docetaxel ferrocenyl derivatives have been designed by replacing the 3′-N-benzoyl group of PTX with a ferrocenoyl moiety, resulting in enhanced anti-proliferative property of the derivative as compared to the parent compound. To synthesize PTX derivatives (**2**), the reaction of optically pure (3R,4S)-3-triethylsilyloxy-4-phenylazetidin-2-one (**3**) (shown in Fig. 4) and 10-deacetyl baccatin III (**1**) with trimethylsilyl chloride in pyridine was employed which was followed by selective *O*-acetylation of 10-OH with LiHMDS and acetyl chloride in THF at − 40 °C for 30 min (Fig. 3).

Further, (3R,4S)-N-Ferrocenoyl-4-phenyl-3-triethylsilyloxyazetidin-2-one (**4**) was synthesized by N-acylation reaction of (**3**) with ferrocenoyl chloride. The N-acylation of (**3**) with 4-ferrocene butyric acid using diisopropyl carbodiimide functioning as a coupling agent with a catalytic amount of 4-dimethylamino pyridine in dichloromethane at room temperature resulted in conversion to N-4-ferrocenyl butyryll-3-triethylsilyloxyazetidin-2-one (**5**) (Fig. 4).

**Fig. 5:**
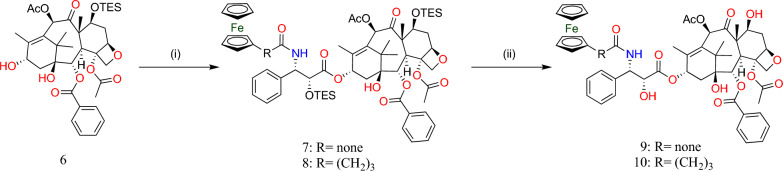
Synthesis of *N*-debenzoyl-*N*-ferrocenoylpaclitaxel derivatives, (9) and (10). (i) LiHMDS, THF, − 40 °C, 40 min; (ii) HF·Py, pyridine/MeCN, room temperature, 24 h

Furthermore, under specific conditions with LiHMDS as a base at − 40 °C, 13-*O*-acylation of (4) (shown in Fig. 4) with azetidine-2-ones (6) and (3) (shown in Fig. 4) produced the corresponding PTXs (7) and (8) derivatives, and deprotection of -OH groups using a greater quantity of HFpyridine produced (9) and (10) PTX analogs (Fig. 5).

**Fig. 6:**
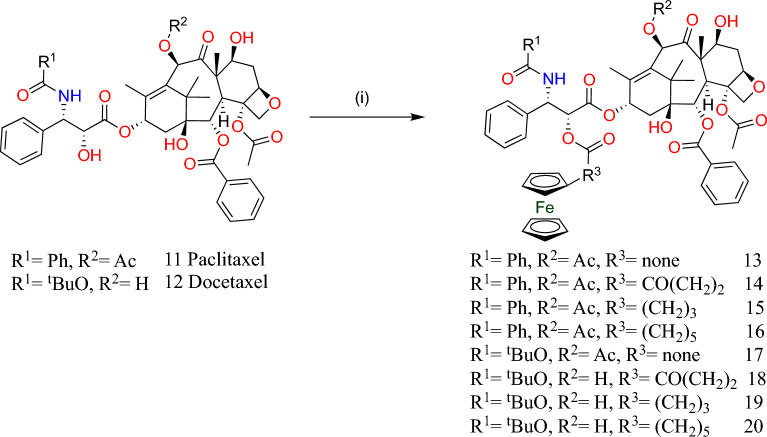
Synthesis of 2′-*O*-ferrocene-substituted paclitaxel 13–16 and docetaxel 17–20 derivatives. Reaction conditions: (i) FcR_3_COOH, DIC (1.5 equiv), DMAP (0.1 equiv), DCM, room temperature, 24 h

Substitution of ferrocenyl moiety linked to the PTX 2′-OH group led to compounds with lower toxicity than that of the PTX. Anti-proliferative activity was decreased on substitution at the 7′-OH group compared to the parent compound. The synthesized derivatives (**16–19**) and (**25–26**) possessed lower activity than the PTX parent compound (Figs. 6, 8).

**Fig. 7:**
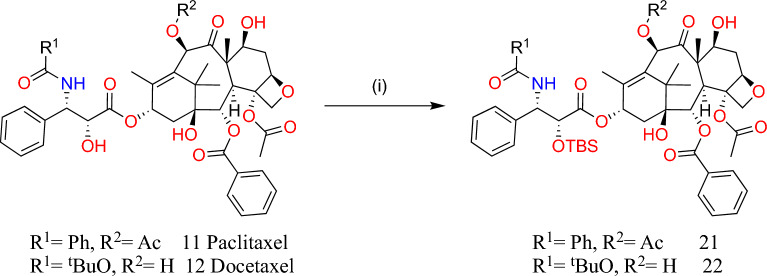
Synthesis of 2′-*O*-TBS ethers of paclitaxel 11 and docetaxel 12. Reaction conditions: (i) TBSCl, imidazole, room temperature, 24 h

For substitution at the 7′-OH position, (**11**) and (**12**) compounds were protected at the 2′-*O*-position as tert-butyldimethylsilyl ethers with tert-butyldimethylsilyl chloride in the presence of imidazole in DMF at room temperature [64]. The compound synthesized (**21**) was selectively 7-*O*-acylated with 3-ferrocenoyl propionic acid or 4-ferrocenyric acid using DIC as a coupling agent at 0 °C resulting in corresponding products **22–23** (Fig. 7).

**Fig. 8:**
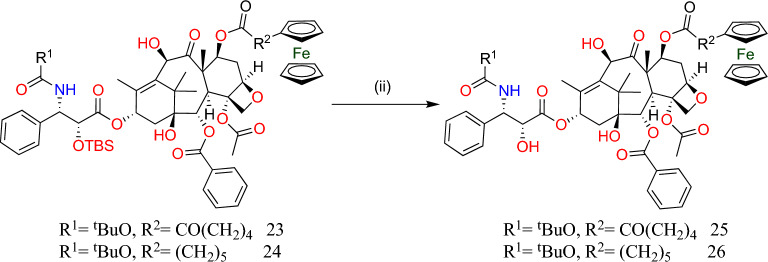
Synthesis of docetaxel derivatives (**25**) and (**26**). Reaction conditions: (ii) HF·Py/pyridine/MeCN, room temperature, 24 h

The 7-*O*-acylated-2′-*O*-TBS-docetaxel derivatives (**23**) and (**24**), shown in Fig. 7, were produced by reacting 2′-*O*-TBS-docetaxel (**22**) (shown in Fig. 7) using 5-ferrocenoylpentanoic and 6-ferrocenylhexanoic acids as acylating agents at 0 °C. Moreover, the deprotection of hydroxy groups was done using HF·Py, which led to the synthesis of the 7-*O*-ferrocenyl-substituted taxanes (**25–26**), respectively [65–68] (Fig. 8).

**Fig. 9:**
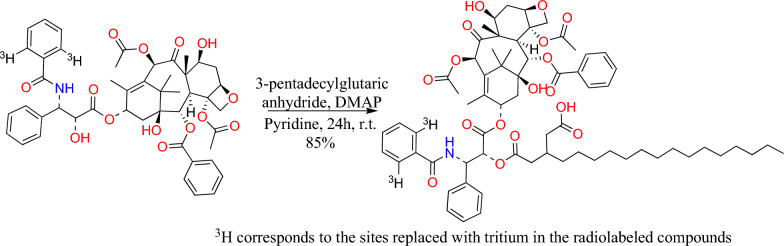
Synthesis of paclitaxel-2′-*O*-3-pentadecylhemiglutarate

[69] prepared a water-soluble ester-linked glucoside derivative of PTX, in which diols anomers of allyl 2,3,4-tri-*O*-benzyl-6-*O*-tritylglycoside were synthesized, followed by their chromatographic separation (Fig. 9).

**Fig. 10:**
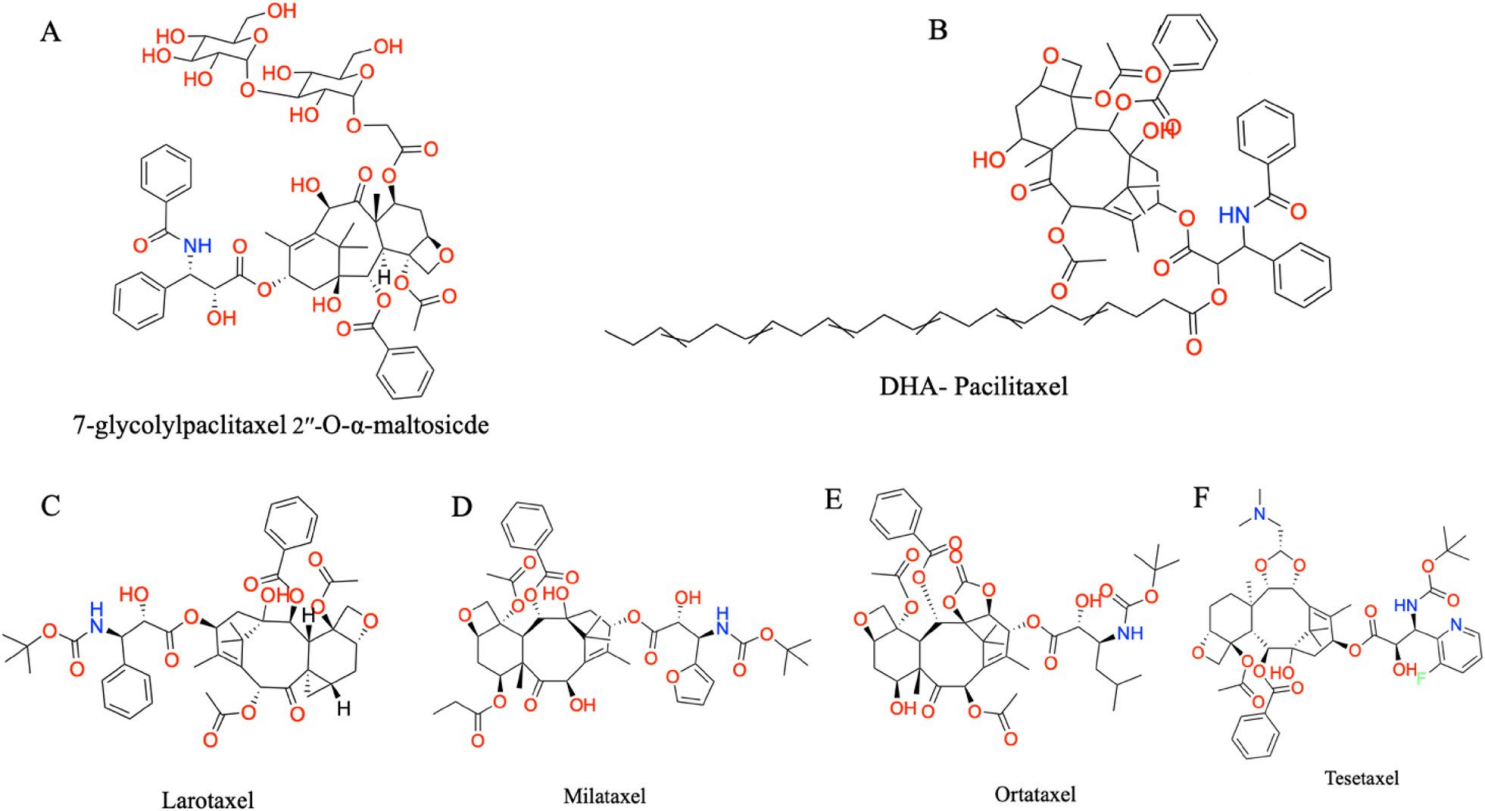
Chemical structure of 7-glycolypacilitaxel (**A**), DHA-paclitaxel (**B**), larotaxel (**C**), milataxel (**D**), ortataxel (**E**), tesetaxel (**F**)

7-glycolyl PTX 2′′-*O*-α-maltoside (Fig. 10a), an ester-linked PTX-glycoside conjugate, has been synthesized by condensing 2′-TES PTX with α-glycosyloxy acetic acid and then deprotecting the hydroxy groups. A PTX dicarboxylic acid derivative was synthesized for specific binding to ubiquitous protein, serum albumin. In the synthesis, hexadecanol was oxidized to palmitaldehyde using PCC and then Wittig olefinated. The synthesized triester was decarboxylated and saponified employing KOH, resulting in an activated anhydride of 3-pentadecyl glutaric acid. The synthesized derivative showed higher cytotoxicity, high serum stability, and efficiency than PTX [70]. Li created NucA-PTX, a water-soluble PTX-nucleolin-aptamer combination, to precisely deliver PTX to the ovarian cancer tumor spot. A more durable and inactive dipeptide bond sensitive to cathepsin B joins the tumor-targeting nucleolin aptamer to the active hydroxyl group at the 2′-position of PTX [71].

Over the past 10 years, taxane liposomal formulations have undergone substantial research and development [72]. For instance, clinical studies employing the PTX-loaded cationic liposome EndoTAG-1 and the PTX-entrapped liposome LEP-ETU have shown promising results. New-generation taxoids carrying DHA at the C2′ position have been generated with the creation of DHA-PTX (Taxoprexin; Fig. 10b). Some of these taxoids showed promising activity against (Pgp +) DLD-1 human colon and (Pgp-) A121 human ovarian cancer xenografts in mice, as well as substantially less toxicity than the parent taxoids in terms of systemic toxicity [73].

The creation of PTX prodrugs has considerably used the C2 position to boost water solubility and anticancer effectiveness. The effects of several PTX conjugates, including docosahexaenoic acid (DHA)-PTX ("Taxoprexin") and poly (l-glutamic acid) PG-PTX ("Opaxio"), are now being researched in humans [74]. In contrast to PTX and DHA-PTX ("Taxoprexin") and PTX, DHA-SB-T-1214 had a significant antitumor impact on mouse xenografts of the DLD-1 human colon, H460 human non-small cell lung, CFPAC-1, and PANC-1 human pancreatic cancer [75].

Currently being evaluated in clinical settings are the PTX analogs larotaxel, milataxel, ortataxel, and tesetaxel (Fig. 10c–f). Larotaxel is being evaluated in clinical studies for metastatic breast cancer, advanced NSCLC, urethral bladder cancer, and advanced pancreatic cancer [76–78]. Larotaxel plus cisplatin did not outperform cisplatin/gemcitabine in a Phase III trial for locally advanced or metastatic bladder or urothelial tract cancer [79]. For NSCLC resistant to taxanes, recurrent glioblastoma, and metastatic breast cancer, Ortataxel is now undergoing phase II research [80]. Tesetaxel has already finished Phase I and II trials in solid tumors [81, 82]. Milataxel was promising in a distinct study of individuals with platinum-refractory NSCLC [83]. However, it was unsuccessful in a Phase II trial for advanced previously treated colorectal cancer. When given at a 60 mg/m^2^ dose, BMS-184476 was well tolerated and effective against NSCLC in patients with previous therapy [84].

## Mechanism of antitumor action of paclitaxel

### Stabilization of the microtubule

**Fig. 11:**
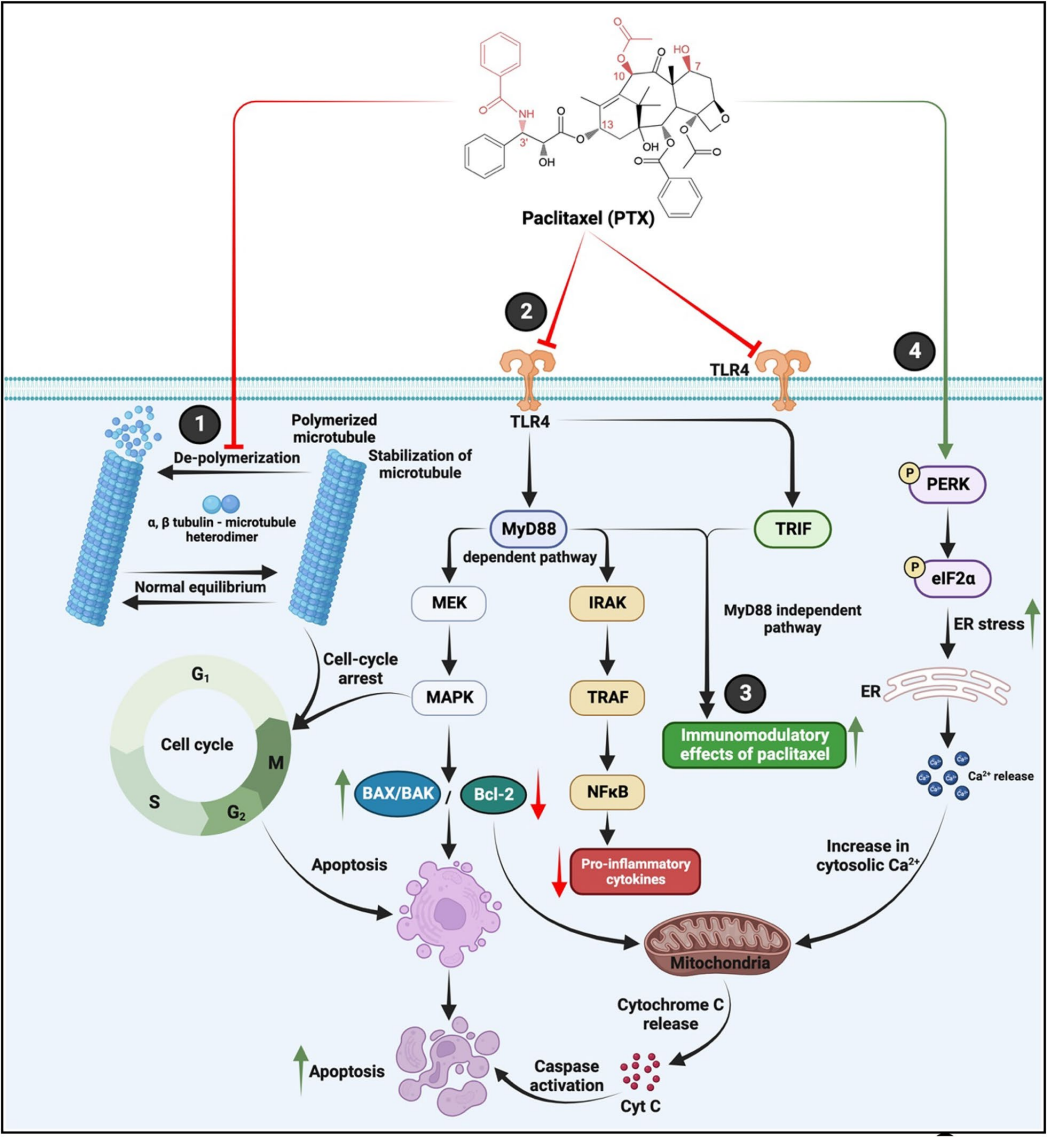
Mechanism of anticancer action of Paclitaxel (PTX): PTX intervention leads to (1) stabilization of microtubule, cell arrest, and subsequent apoptosis, (2) inhibition of the TLR4 signaling pathway, (3) increase in the immunomodulatory effects of the drug and (4) activates ER stress-mediated cell death in different cancers. BAK: Bcl-2 homologous antagonist/killer; BAX: Bcl-2 associated X protein; Bcl-2: B cell lymphoma 2; Cyt C: cytochrome C; eIF2α: eukaryoic translation initiation factor 2 alpha; ER: endoplasmic reticulum; IRAK: interleukin 1 receptor-associated kinase; MAPK: mitogen-activated protein kinase; MEK: MAPK/extracellular signal-regulated (ERK) kinase; MyD88: myeloid differentiation primary response protein 88; NFκB: nuclear factor light chain enhancer of kappa; PERK: PRKR-like endoplasmic reticulum kinase; PTX: paclitaxel; TLR4: toll-like receptor 4; TRAF: tumor necrosis factor (TNF) receptor-associated factor; TRIF: TIR domain-containing adaptor protein. Created with Biorender.com

Paclitaxel (PTX) primarily targets the microtubules (microtubule targeting agent; MTA), the cytoskeletal architecture of the cells, that play critical roles in cellular processes such as cell cycle progression and division, motility, and intracellular trafficking [33, 85, 86]. The net microtubule assembly rate equals the net disassembly rate during steady-state conditions, and the length of the microtubule is unchanged [33]. Microtubules are assembled from α- and β-tubulin heterodimers in a head-to-tail pattern (rapidly growing '+ve end' at one side and slower growing '−ve end' at the other) during the G_2_ phase and the prophase of mitosis and its disassembly (a process called dynamic instability) requires GTP hydrolysis [33, 85]. PTX blocks depolymerization of the microtubule during cell cycle progression, specifically by binding to the N-terminus of the β-tubulin subunit, thereby stabilizing the polymerized microtubule, causing cell cycle arrest at G_2_/M-phase, causing non-progression of the cell cycle and subsequently leading to apoptosis [86–89] [Fig. 11-(1)].

Interestingly, PTX stabilized microtubule formation in vitro and was resistant to low-temperature or calcium-triggered depolymerization [34, 90]. PTX intervention caused mitotic arrest in cell cultures and animal tumor models [34]. The findings suggest that PTX-arrested cells in the metaphase and the cells maintain near-normal bipolar spindles [32]. Studies have also revealed that low PTX concentrations blocked the depolymerization of microtubules. In contrast, at higher concentrations, it increased the stability of microtubules and inhibited the separation of microtubule '-ve' ends from centrosomes [91–93]. The efficacy of PTX as an MTA is reduced when cells confer resistance to the drug through premature mitotic exit (mitotic slippage), thus evading the PTX-induced cell cycle arrest and subsequent apoptosis [94].

### Effect on the TLR4 pathway

Concentration and time-dependent anticancer and pro-apoptotic effects of PTX, independent of its effect on microtubule depolymerization, have been reported. PTX modulates the transcription of several genes, directly or indirectly involved in cell proliferation, apoptosis, and inflammation via dysregulation of the TLR4 (toll-like receptor 4) pathway, which in turn can be either via MyD88 (myeloid differentiation primary response protein 88) dependent and/or independent pathways [95]. In normal cells, the TLR4 pathway plays an integral role in cellular defense/survival mechanisms, pathogen recognition, and pattern recognition, activating innate immunity and eliciting immune responses [96–98]. Active TLR4 signaling, initially identified in breast cancer cells, has been implicated in the chronic inflammation-mediated development of different cancers, cancer progression, chemotherapeutic resistance, cancer cell stemness, invasion, metastasis, and disease relapse [95, 99–102]. The activation of TLR4 mediated MyD88-dependent pathway subsequently activates several pro-oncogenic and anti-apoptotic signaling mechanisms in cancer cells that include the Raf1/MEPK/MAPK pathway (associated with cell survival) and the IRAK/TRAF/NFκB pathway (associated with the synthesis and secretion of pro-inflammatory cytokines) [86, 98] [Fig. 11-(2)]. The MyD88-dependent pathway also activates the MAPK pathway in cancers that contribute to cancer cell proliferation, resistance to programmed cell death, and synthesis of pro-inflammatory cytokines (via AP-1 activation) [103, 104] [Fig. 11-(2)]. The TRIF (MyD88-independent) mediated mechanism of the TLR4 pathway results in the phosphorylation and activation of transcription factor IRF3, which then translocates to the nucleus and drives the expression of Type 1 interferons [98].

**Fig. 12:**
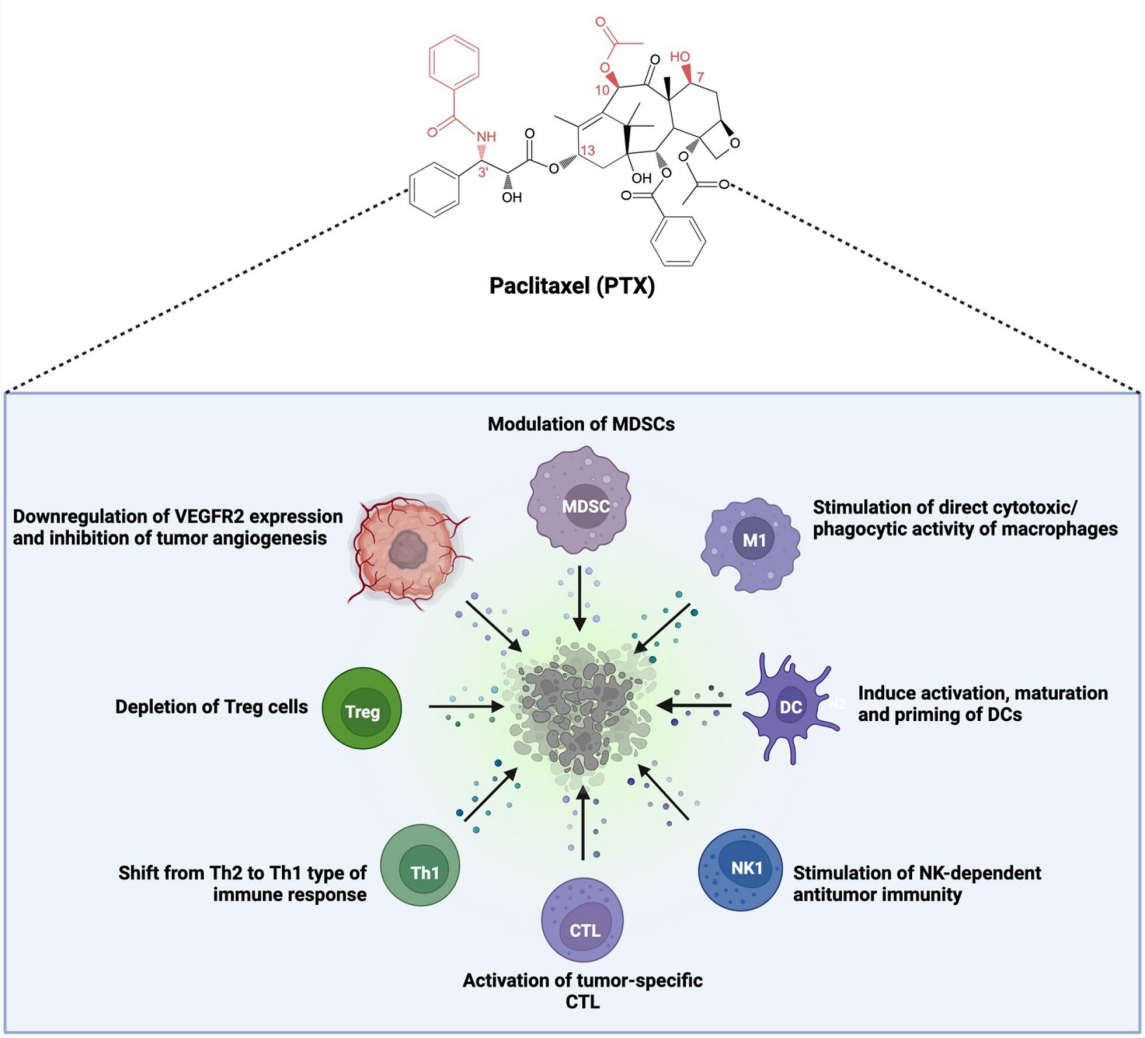
Antiangiogenic and immunomodulatory effects of Paclitaxel (PTX): PTX exerts its anticancer activity via its antiangiogenic and immunomodulatory effects. CTL: cytotoxic T-lymphocyte; DC: dendritic cells; M1: M1 macrophages; MDSC: myeloid-derived suppressor cells; NK1: natural killer type 1 cells; Th1: Type 1 T-helper cells; Treg: regulatory T cells; VEGFR2: vascular endothelial growth factor receptor 2. Adapted and modified from Kampan et al., 2015 [33]. Created with Biorender.com

PTX intervention effectively blocks the MyD88-dependent and independent cancer-promoting TLR4 mechanisms and trigger apoptosis (via upregulation of pro-apoptotic BAX/BAK and downregulation of anti-apoptotic Bcl-2), reduce chronic inflammation, and activate several immunomodulatory tumor combating effects of PTX (Figs. 11-(3) and 12) [33, 86].

However, PTX-mediated modulation of the TLR4 pathway is implicated in the activation of NF-κB and MAPK-related downstream signaling and subsequent release of pro-inflammatory molecules that enhance the progression of cancers and confer chemoresistance to drug intervention in cancers [105, 106]. PTX at lower doses induces cytokines and pro-inflammatory proteins and apoptosis at higher doses. Hence, the dose-dependent effects of PTX in different cancers must be carefully addressed to maximize the anticancer efficacy of the drug [107].

### Paclitaxel-mediated activation of ER stress

Certain studies have implicated the PTX-mediated activation of the endoplasmic reticulum (ER) stress response or the unfolded protein response (UPR) via the PERK and IRE1α [Fig. 11-(4)], subsequent apoptotic cell death in different cancers [108, 109]. PTX chemotherapy triggers the activation of antitumour immunity through immunogenic cell death via TLR4 and enhances the expression of CALR mediated by CCL2 transcription and IκB kinase-2 SNARE-dependent exocytosis resulting in activation of NF-κB signaling pathway [108]. Combinational therapy of apatinib and PTX induces ER stress, autophagy and apoptosis in ECA-109 and KYSE-150 esophageal squamous cancer cells (ESCC). Further, combination of apatinib and chloroquine enhances the sensitivity in ESCC which in turn triggers PTX for apoptosis through IRE-1α-AKT-mTOR signaling pathway [109]. In conclusion, PTX in combination with other drugs mediates the ER stress response via PERK, IRE1α and NF-κB signaling pathway resulting in apoptosis and immunogenic cell death of tumor cells.

### Other anticancer effects of paclitaxel

Several studies have reported the solid angiogenic inhibitory activity of PTX at low doses by modulating VEGF expression and the VEGF signaling pathway by downregulating VEGFR2 [33, 110]. One of the mechanisms of action of PTX involves tumor cell death via the generation of reactive oxygen species (ROS) [86]. The combinational therapy of PTX with glucose inhibitors, such as 2-deoxy D-glucose and hydro peroxide (L-buthionine-S, R-sulfoximine), selectively increased hydrogen peroxide mediated breast cancer cell death [111].

There are many challenges in understanding the detailed and precise action of PTX and the specific relevant concentration for using PTX in cell culture. A probable reason could be varying usable concentrations of PTX in diverse cancer types and chemotherapy-associated interventions. The changing PTX levels in plasma and excess PTX accumulation in cell lines reflect greater concentration in cells than in plasma; hence, it is not easy to measure its effective concentration [32]. Instinctively, how PTX interferes specifically at interphase without affecting prior mitosis is still unclear. Some authors hypothesized that PTX might have interfered with cell signaling and microtubule-mediated transport. The microenvironment of cell culture has a profound effect on PTX antitumor activity, e.g., in the context of drug testing, clinically relevant amounts of PTX do not cause death in cells at interphase and have not gone through mitosis [112].

### Scientific studies on the antitumor effect of PTX

Non-small-cell lung cancer (NSCLC) is one of the most common cancers in the USA, accounting for 85% of lung cancer cases. In 1999, PTX was recognized as an FDA-approved drug for NSCLC. Mohiuddin et al. examined the underlying mechanisms of PTX's inhibitory effect on gefitinib-resistant NSCLC cells (PC9-MET). The results demonstrate that PTX significantly reduced the PC9-MET cell viability and apoptosis induction. The apoptotic impact was also accompanied by enhanced cleaved caspase-3, 9, and PARP levels. PTX augmented oxidative stress by enhancing ROS production, which in turn caused DNA damage in tumor cells. PTX eliminated cellular senescence related to the inactivation of p53/p21 and p16/pRb signaling pathways. The authors concluded that PTX is a hopeful antitumor drug offering a new therapeutic approach for managing gefitinib-resistant NSCLC during the COVID-19 pandemic [113].

In another study, using a time-dependent approach, PTX nanoparticles loaded with polylactic-co-glycolic acid were employed to observe their antitumor effect on NSCLC cells in vitro. The authors demonstrated that PTX nanoparticles inhibited A549 and H1650 cell activity. Although the inhibitory activity was less at 12 and 24 h with the progression of time, a potent inhibition occurred at 48 and 72 h. The nanoparticles were more effective in triggering apoptosis, blocking invasion, and migrating NSCLC cells than normal PTX. The sustained release with more efficient cellular uptake made PTX nanoparticles a hope as a promising drug carrier in lung carcinoma [114].

The PTX chemotherapy is extensively implemented to manage several tumors listed as breast, ovarian, and NSCLC. Nowadays, combinational therapy has been effective, overcoming many challenges associated with single-drug chemotherapy. Kim et al. [115] employed a combinational approach to study the antitumor activity of PTX with sorafenib and radiation in vitro and in vivo in anaplastic thyroid cancer (ATC) cells. The authors concluded that a combination of synergistically in vitro lowered the cell viability of tumor cells and increased cell apoptosis. The xenograft model reported a significant decline in tumor volume and enhanced survival rate, representing it as a potential therapy in preclinical models.

Another combinational therapy by Khing et al. [116], in which PTX was given in adjunction with fluoxetine, was evaluated for antitumor activity in gastric adenocarcinoma cells. The combination resulted in the G2/M-phase arrest and triggered early and late cell death plus necroptosis in a time and dose-dependent fashion.

[117] studied the caffeic acid and PTX (in combination) effect on NSCLC cells both in vivo and in vitro. Co-treatment showed that caffeic acid enhanced the cytotoxicity of PTX in H1299 cells at low concentrations but not in Beas-2b cells.

The cells H1299 were arrested at the sub-G1 phase and triggered caspase-3, 9 followed by apoptosis. Caffeic acid improved the phosphorylation of c-Jun NH2-terminal protein kinase1/2, Bax, and Bid, and their activation. Additionally, in vivo, study reported that PTX and caffeic acid suppressed the tumor growth in the H1299 xenograft model without any adverse effects.

The synergistic influence of silibinin and PTX on ovarian cancer has been investigated by [118] in ovarian carcinoma cell lines SKOV-3. Results revealed a considerable slowing of the SKOV-3 cells' development followed by induction of apoptosis. Tumor suppressor genes p53 and p21 upregulation is reported along with a crosswalk between PTX, silibinin, and cancer via computational analysis.

In human prostate cancer, a combination of PTX and noscapine was analyzed for antitumor properties in vitro. The tumor cells’ viability declined, improved apoptosis, decreased expression of Bcl-2, and increased Bax and Bcl/Bax ratio LNCaP and PC-3 cells. The expression of androgen receptor and prostate-specific antigens declined in LNCaP cell lines [119]. Han et al. [120] reported that a combination of PTX and ruxolitinib synergistically enhanced the antitumor property of an antineoplastic agent and suppressed tumor growth in the human ovarian mouse model. However, a recent study on ovarian cancer cell lines MES synergistically demonstrating the effect of low-dose PTX with *Asparagus officinalis* revealed that the combination of congested cell proliferation and cell invasion triggered apoptosis. The mechanism of action was DNA-dependent damage, suppression of microtubule dynamics and associated proteins, and AKT/mTOR pathway interference [121]. Trastuzumab, a humanized anti-human epidermal growth factor receptor 2 antibody drug, when given in combination with PTX enhanced the antitumor efficacy of trastuzumab-resistant in resistant and sensitive xenografted tumors. The combination resulted AKT–p27^kip1^–retinoblastoma protein pathway and apoptosis [122].

Researchers have also prepared nano-formulations of PTX to enhance the drug's bioavailability, specificity, and antitumor activity in different types of cancers. Huang et al. [123] encapsulated PTX with PEG-PLA/TPGS and found the PTX-micelles to improve the anticancer property of PTX in A549 non-small lung cancer cells. The xenograft model studies on nude mice revealed that PTX micelles could block tumor growth more efficiently than other formulations. Leiva et al. [124] formulated PTX nanoparticles with glyceryl tripalmitate (tripalmitin), including adjustments by adding hexa (ethylene glycol), β-cyclodextrin, and macelignan. All the nano-formulations reported excellent hemocompatibility and improved antitumor activity in breast and lung cancer cells. Tripalm-NPs-PC declined IC_50_ by 40.5 and 38.8-fold in breast and lung cancer, respectively. Moreover, the exact formulation reduced the breast volume and lung multicellular tumors. The authors concluded that Tripalm-NPs-PC enhanced the antitumor property and is an alternative and practical PTX delivery system in lung and breast cancer.

## Traditional medicine versus standard clinical practice: current medical applications

**Fig. 13:**
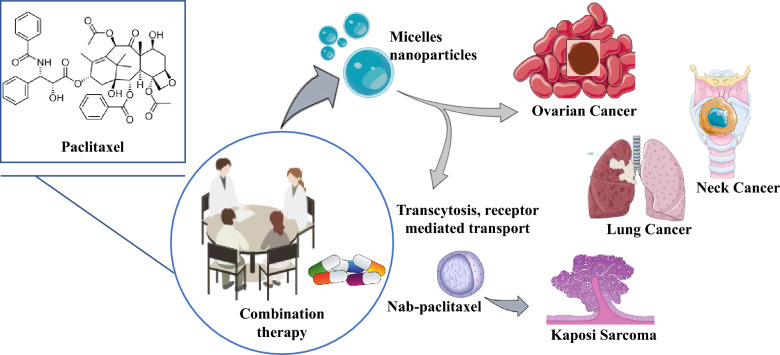
Paclitaxel vital role in several cancer types

The discovery of PTX was a significant breakthrough in cancer treatment. Today, PTX is commonly used as a chemotherapy drug and is available in various formulations for different administration routes, including intravenous and oral administration. PTX has played a vital role in cancer treatment and has significantly improved patient outcomes in several cancer types (Table 1 and Fig. 13). It is the principal taxane-derived antineoplastic drug used in the cancer treatment [125]. The PTX detection involved screening 35,000 medicinal plants by U.S. National Cancer Institute (1958) for cytotoxic efficacy. Later, in 1971, it was extracted from the *Taxus brevifolia* bark [126]. PTX is regarded as a gold standard chemotherapeutic drug for healing different cancer types, such as ovarian, breast, urothelial, head and neck, Kaposi's sarcoma, and non-small cell lung carcinoma [127, 128]. To enhance its therapeutic efficacy and overcome limitations, novel drug formulations incorporating PTX, such as polymeric micelle nanoparticles, have been developed and applied in anticancer curing regimes. PTX also exhibits radiation-sensitizing effects.

**Table 1:** Medical applications of paclitaxel for curing many diseases

Plant species	Preparation/extraction form	Mode of administration	Diseases cured	Country	References
*Taxus brevifolia*	Paclitaxel	In vivo	Ovarian cancer	USA	[131]
*Taxus brevifolia*	Paclitaxel	In vivo	Breast cancer	USA	[132]
*Taxus brevifolia*	Paclitaxel	In vivo	Lung cancer	USA	[133]
*Taxus brevifolia*	Paclitaxel	In vivo	Kaposi’s sarcoma	USA	[134]

The discovery of PTX from plant screening led to its inclusion in clinical trials, and it became the only plant-derived drug to be enlisted in such trials [129]. A unique mechanism of PTX action, targeting microtubule assembly, was discovered in 1979 and approved by the Food and Drug Administration in 1992 and 1994 for ovarian and breast cancer [129]. At the moment, PTX is used individually or in combination with supplementary medications to treat breast, ovarian, and non-small cell lung cancer [129, 130].

PTX is a potent anticancer drug frequently utilized in ovarian cancer prevention, both in adjuvant and advanced settings, often administered in combination with additional chemotherapy drugs like carboplatin and doxorubicin or cyclophosphamide. The usage of PTX in ovarian cancer treatment has been well-established and is supported by several clinical trials and guidelines [131]. PTX-based regimens have been extensively studied and recommended as a standard option for the prevention of breast as well as metastatic cell lung cancer [132]. It is often administered in amalgamation with platinum-based chemotherapy drugs like cisplatin or carboplatin. PTX-based regimens were evaluated in clinical trials and are commonly recommended for the management of the NSCLC [133]. PTX has demonstrated activity against Kaposi sarcoma, a type of cancer often occurring in people with debilitated immune systems, like HIV/AIDS. It can also be used alone or with other medications to treat Kaposi sarcomas [134].

As a chemotherapeutic agent, it can be practiced to cure various cancers like breast, ovarian, lung, pancreatic, and solid tumors. While PTX is an effective chemotherapeutic agent, it can cause several adverse effects, including decreased red blood cells, white blood cells, and platelets (bone marrow suppression), peripheral neuropathy, myalgia (muscle pain), arthralgia (joint pain), gastrointestinal disturbances, and hair loss [135].

## PTX derived from endophytes of different plant species for improved production

The challenges associated with sluggish plant growth (*Taxus*) and the limited PTX yield have prompted the search for alternative strategies for PTX production. Over the past 40 years, various biotechnological approaches have been developed to address these challenges. These approaches include field cultivation, chemical synthesis, cell suspension, callus, hairy root, and tissue culture [136–138]. While these biotechnological methods have shown promise, they have not been widely adopted for large-scale PTX production due to several limitations. These limitations include massive reaction steps, lengthy incubation times, and low yields, which make these methods impractical for meeting the increasing demand for PTX. Researchers have been working to overcome these limitations and develop more efficient and scalable strategies for PTX production [139–141].

In recent years, endophytes have gained the attention of researchers for their potential in PTX production. Endophytes can be sequestered from various host plant species belonging to different families. They are in diverse ecological and geographical conditions, indicating their adaptability to various environments. Endophytes possess the genetic machinery necessary for PTX biosynthesis and modulate the gene expression in secondary metabolite biosynthesis pathways. Endophytes can potentially overcome the limitations associated with traditional methods of PTX production, such as low yields and long incubation times. However, the exploration of endophytes for PTX production is still in its early stage, and vital research efforts are required to harness their potential fully [142].

Researchers have discovered over 35 species of endophytic fungi proficient in producing PTX. Some of these species include *Glomerella cingulata, Pestalotiopsis terminaliae, Fusarium oxysporum, Nigrospora sphaerica, Lasiodiplodia theobromae, Colletotrichum gloesporioides, Phyllosticta tabernaemontanae, Pestalotiopsis microspora, Chaetomella raphigera, Alternaria alternata,* and *Cladosporium oxysporum* [143, 144]. PTX can also be created by endophytes connected to a variety of other plant groups in addition to those belonging to the Taxaceae family, which are known for producing the drug, such as *Rubiaceae, Rutaceae, Rhizophoraceae, Solanaceae, Sapindaceae, Plantaginaceae, Podocarpaceae, Pinaceae, Malvaceae, Magnoliaceae, Moraceae, Lamiaceae, Ginkgoaceae, Combretaceae, Cupressaceae, Acanthaceae* and *Araucariaceae* [144].

Several species of *Aspergillus,* including *A. terreus, A. fumigatus, A. niger, A. aculeatinus* and *A. oryzae*, have been specified as PTX producers. Notably, *A. fumigatus* is confirmed as a high PTX-producing species on the S7 medium. Other PTX-producing fungi includes *Beauveria sp., Mycelia sterilia, Epicoccum sp., Fusarium sp., Stemphylium sedicola, Alternaria sp., Cladosporium sp.* and *Paraconiothyrium variabile.* These fungi have been sequestered from diverse host plants and demonstrated PTX production under specific culture conditions [145] (Table 2).

**Table 2:** Paclitaxel isolated from endophytes of different host plant species

Plant Species	Plant parts	Extraction solvents	Methods of detection	Endophytic fungi	Country	References
*Taxus chinensis* var. *mairei*	Twig, old inner bark	Methylene chloride	LCMS, HPLC, CIEIA	*Didymostilbe* sp.	China	[160]
*Taxus chinensis* var. *mairei*	Bark	Dichloromethane	ESI–MS, HPLC	*Aspergillus aculeatinus*	China	[145]
*Taxus baccata* L	Wood		LC–MS, HPLC, EIA	*Alternaria* sp.	Italy	[161]
*Taxus baccata* L	Twigs	Dichloromethane	HPLC, LC–MS/MS	*Paraconiothyrium variabile*	U.K	[162]
*Taxus baccata* L	Yeast extract	Yeast extract	HPLC	*Cladosporium* sp.	Iran	[163]
*Taxus baccata* L. subsp. *Wallichiana* (Zucc.)	Bark	Methanol	HPLC–MS	*Fusarium redolens*	India	[164]
*Taxus brevifolia* Nutt	Inner bark	Dichloromethane	HPLC, MS, TLC	*Taxomyces andreanae*	Northern Montana	[165]
*Taxus celebica* (*Warb.*) H.L. Li	Stem	Methylene chloride	LC–ESI–MS, TLC, HPLC	*Fusarium solani*	UK	[166]
*Taxus chinensis* Roxb	Bark	Ethyl acetate	LC–MS, ESI–MS, HPLC	*Metarhizium anisopliae*	China	[167]
*Taxus chinensis* Roxb	Bark	Ethyl acetate	HPLC	*Fusarium solani*	China	[168]
*Taxus chinensis* Roxb	Bark	Dichloromethane	LC–MS, ELISA	*Mucor rouxianus* DA10	China	[169]
*Taxus cuspidate* Sieb. & Zucc	Leaves	Dichloromethane	LC–MS, NMR HPLC, UV, IR	*Phomopsis* sp.	South Korea	[170]
*Taxus cuspidate* Sieb. & Zucc	Inner bark	Ethyl acetate, Methanol	LC–MS	*Aspergillus niger*	China	[171]
*Taxus cuspidate* Sieb. & Zucc	Inner bark	Chloroform/methanol	TLC, NMR, RP-HPLC	*Fusarium arthrosporioides*	Korea	[172]
*Taxus mairei* (Lemée & H.Lév.)	Bark	Diethyl sulfate Chloroform, methanol	MS, CIEIA, HPLC	*Fusarium maire*	China	[173]
*Taxus mairei* (Lemée & H.Lév.)	Inner bark	Chloroform/methanol	MS, HPLC, UV, TLC	*Tubercularia* sp.	China	[174]
*Taxus* × *media Rehder*	Bark, needles	Ethyl acetate	HPLC–MS	*Guignardia mangiferae*	China	[175]
*Taxus* × *media Rehder*	Inner bark	Chloroform, methanol	NMR, HPLC	*Cladosporium cladosporioides*	Canada	[176]
*Taxus* × *media Rehder*	Bark	Chloroform/methanol	LC–MS	*Aspergillus terreus*	Canada	[177]
*Taxus wallichiana* Zucc	Inner bark	Methylene chloride	NMR, MS	*Pestalotiopsis microspora*	India	[178]
*Taxus wallichiana* Zucc	Stem	Methylene chloride	LC–MS, TLC	*Sporormia minima*	Nepal	[179]
*Aegle marmelos* Correa ex Roxb	Leaves	Methylene chloride	HPLC, TLC, UV	*Bartalinia robillardoides*	India	[180]
*Taxus wallichiana* var*. mairei*	Bark	Chloroform, methanol	ESI–MS/MS, HPLC	*Phoma medicaginis*	China	[181]
*Calotropis gigantea* (L.) R. Br	Leaves	Dichloromethane	HPLC, FTIR	*Phoma* sp.	India	[182]
*Capsicum annuum* L	Fruit	Dichloromethane	HPLC	*Colletotrichum capsici*		[170]
*Cardiospermum halicacabum* L	Leaves	Methylene chloride	HPLC	*Pestalotiopsis pauciseta*		[180]
*Citrus medica* L	Leaves	Dichloromethane	HPLC, NMR	*Phyllosticta citricarpa*	India	[146]
*Corchorus olitorius* L	Leaf, flower, seed, stem, root	Ethyl acetate	FTIR, LC–ESI MS/MS, TLC, HPLC	*Grammothele lineata*	Bangladesh	[183]
*Cupressus* sp*.*	Needles	Dichloromethane	UV, IR, TLC, HPLC	*Phyllosticta spinarum*	India	[146]
*Ginkgo biloba* L	Leaves	Dichloromethane	UV, IR, HPLC,	*Phomopsis* sp*.*	South Korea	[170]
*Hibiscus rosasinensis* L	Leaves	Dichloromethane	HPLC	*Phyllosticta dioscoreae*	India	[184]
*Justicia gendarussa* Burm. f	Leaves	Methylene chloride	HPLC	*Colletotrichum gloeosporioides*	India	[185]
*Larix leptolepis* L	Leaves	Dichloromethane	HPLC, LC–MS, NMR, UV, IR	*Phomopsis* sp*.*	South Korea	[170]
*Michelia champaca* L	Needles	Dichloromethane	HPLC, UV	*Chaetomium* sp.	India	[186]
*Moringa oleifera* Lam	Leaves	Dichloromethane	LC–MS, IR, NMR, UV, HPLC	*Cladosporium oxysporum*	India	[152]
*Morinda citrifolia* L	Leaves	Dichloromethane	HPLC, NMR, UV, IR, FAB-MS	*Lasiodiplodia theobromae*	India	[148]
*Plantago major* L	Leaves	Ethyl acetate extract	LC–MS, UV	*Nigrospora sphaerica*	India	[187]
				*Colletotrichum gloesporioides*		
				*Alternaria alternata*		
				*Glomerella cingulate*		
*Salacia oblonga* Wall	Bark		Genomic mining	*Armilaria* sp.	India	[188]
				*Phoma* sp.		
				*Fusarium* sp.		
				*Alternaria* spp. *Pho Coriolopsis*		
				*caperata*., *mopsis* sp.		
				*Lasiodiplodia theobromae*		
				*Trichoderma longibrachiatum*		
				*Botryosphaeria rhodina Aspergillus terreus*		
*Rhizophora annamalayana*	Leaves	Ethyl acetate	TLC, IR, HPLC	*Fusarium oxysporum*	India	[189]
*Plectranthus Amboinicus (Lour.)*	Leaves	Dichloromethane	TLC, UV	*Pestalotiopsis microspora*	India	[149]
*Tarenna asiatica* (L.)	Leaves	Dichloromethane	LC–MS, FTIR, UV–Vis, TLC	*Aspergillus oryzae*	India	[154]
*Taxodium distichum* (L.)	Bark	Dichloromethane	HPLC, TLC, UV	*Aspergillus fumigatus*	Egypt	[151]
*Terminalia arjuna* (Roxb. ex DC.)	Bark	Dichloromethane	HPLC, TLC, UV	*Alternaria tenuissima*	Egypt	[151]
*Terminalia arjuna* (Roxb. ex DC.)	Needles	Ethyl acetate	FTIR, LC–ESI–MS, HPLC, UV	*Alternaria brassicicola*	India	[190]
*Terminalia arjuna* (Roxb. ex DC.)	Leaves	Methylene chloride	FAB-MS, NMR, UV, IR	*Chaetomella raphigera*	India	[17, 191]
*Terminalia arjuna* (Roxb. ex DC.)	Leaves	Methylene chloride	UV, TLC, HPLC	*Pestalotiopsis terminaliae*	India	(Venkatraman Gangadevi & Johnpaul Muthumary, 2009b)
*Wrightia tinctoria* (Roxb.)	Leaves	Dichloromethane	HPLC	*Phyllosticta tabernaemontanae*	India	[146]
*Torreya grandifolia* Raf	Inner bark	Methylene chloride	UV, TLC, EIA	*Periconia* sp.	China	[192]

To test the activity, studies by [146, 147] suggest that low concentrations of PTX derived from endophytic fungi can effectively inhibit cell proliferation during mitosis by stabilizing the spindle fibers. It promotes cell death in various cancer cell lines, encompassing lung (HL251), breast (MCF-7, BT220), intestine (Int4070), colon (H116), and leukemia (HLK210) [148] (Table 3).

**Table 3:** Anticancer properties of paclitaxel derived from endophytes through in vitro study

Host plant	Endophytic fungi	Paclitaxel concentration	Cell lines	Incubation time	Results	Country	Reference/s
*Cupressus* sp.	*Phyllosticta spinarum*	0.005 to 5 mM	HLK 210, HL 251, BT220, Int 407	Apoptotic assay (48 h)	At 0.005–0.05 μmol L^−1^, increase in cell death, and at 0.05–0.5 μmol L^−1^, slightly increase in cell death, while 0.5–5 μmol L^−1^ decreased the cell death	India	[146]
*Taxus chinensis* (Pilg.)	*Mucor rouxianus*	Liver carcinoma	–	24 h	5.2 *} 1.6 × 10–3 mg/m was ED_50_ for the fungal taxol	China	[169]
*Cardiospermum halicacabum* L	*Pestalotiopsis pauciseta*	0.005–0.5 μmol/L	H116, BT 220, HL 251, HLK 210 and Int 407	Apoptotic assay	0.005–0.05 μmol L^−1^, increased in cell death; 0.05–0.5 μmol L^−1^, slightly increased the cell death Whereas 0.5–5 μmol L^−1^, decreased the cell death	India	[147]
*Taxus mairei*	*Tubercularia* sp.		KB, P388	MTT assay	Polymerization of tubulin induced	China	[174]
*Aegle marmelos* (L.)	*Bartalinia robillardoides*	0.005–5 μM	HL 251, HLK 210, H116, Int 407, BT 220,	Apoptotic assay (24 h, 48 h & 72 h)	Morphological changes in cancer cells	India	[180]
*Ginkgo biloba* L	*Phoma betae*	0.005 to 0.05 μM	ATCC HTB-22, T98G, ATTCC CRL-1690, MCF-7, A549, ATCC CCL-185	Apoptotic assay (48 h)	At 0.005–0.05 μmol L^−1^, cell death was increased while at 0.5–5 μmol L^−1^, cell death was decreased	South Korea	[193]
*Capsicum annuum* L	*Colletotrichum capsica*	0.005–0.5 μmol/L	HL 251, HLK 210 and MCF-7	Apoptotic assay (48 h)	0.005–0.05 μmol L^−1^, increased in cell death; 0.05–0.5 μmol L^−1^, slightly increased the cell death Whereas 0.5–5 μmol L^−1^, decreased the cell death	South Korea	[170]
*Tarenna asiatica* (L.)	*Aspergillus oryzae*	30 μg/mL	NCI-H460	MTT assay (6, 12, 24, 48 h)	Shrinkage in cancer cells. Octagonal cells altered into sphere-shaped cells	India	[154]
*Morinda citrifolia* L	*Lasiodiplodia theobromae*	100 to 600 μg/mL	MCF-7	MTT assay (24, 48, 72 h)	Decreased cell viability	India	[148]
*Corchorus olitorius* L	*Grammothele lineata*	0.005 μM	HeLa	24 h	Cell death 35%	India	[183]
*Taxodium distichum* (L.) Rich.*,*	*Aspergillus fumigatus,*	0.39 μg/mL	MCF-7& HepG-2	MTT assay (24 h)	Paclitaxel concentration increased	Egypt	[151]
*Plectranthus amboinicus* (Lour.)	*Pestalotiopsis microspora* EF01	0.005 to 0.05 μM	Hep G2	MTT assay	Showed the vigorous cytotoxic activity towards human liver carcinoma cell lines (Hep G2)	India	[149]
*Aegle marmelos* (L.)	*Bartalinia robillardoides*	0.0005–5 μM	HL251, BT220, H116, HLK 210, Int407	Phosphatidylserine detection assay (24, 48 and 72 h)	After the 72 h, segmented nuclei degraded cells are blocked in the G2/M-phase	India	[194]
*Calotropis gigantea* (L.)	*Phoma* sp.	100 μg/mL	MCF 7	MTT assay (24 h)	Against MCF 7 cell lines showed strong anti-proliferative activity	India	[182]
*Moringa oleifera* Lam	*Cladosporium oxysporum*	1–7.5 μM	HCT 15	MTT assay (24 h)	Cells becomes shrinked and spherical	India	[152]
*Taxus celebica* (Warb.)	*Fusarium solani*	HepG2, JR4-Jurkat, Ovcar3 HeLa, T47D,	HeLa, HepG2, Jurkat, Ovcar3, T47D	Flow cytometry (24 and 48 h)	Bcl2-overexpressed in J16 Jurkat cells, while the DNA fragmentation, and stimulation of caspase-10 in JR4-Jurkat cells	India	[153]
*Tabebuia pentaphylla* Hemsl	*Tabebuia pentaphylla* Hemsl	100 μg to 700 μg	MCF-7	MTT assay (24, 48 and 72 h)	Spherical in shape	India	[150]
*Taxus chinensis* var. *mairei*	*Didymostilbe* sp.	50 μg/mL	BEL7402	48 h	Little cytotoxicity	China	[195]

Rajendran et al. [149] extracted endophytic fungi (*Pestalotiopsis microspore*) from *Plectranthus amboinicus*, producing a better yield of PTX, which showed cytotoxic activity against the Hep G2 cell line. The PTX effects (*Pestalotiopsis pauciseta*) on the growth of MCF-7 breast cancer cells were examined by [150]. They observed that PTX exhibited a higher cytotoxic effect on MCF-7 cells. Ismaiel et al. [151] identified *A. tenuissima* and *A. fumigatus* isolated from *Terminalia arjuna and Taxodium distichum* as producers of PTX. Various human cancer cell lines (HepG-2, A-549, MCF-7, CHO-K1, and HEp-2) were subjected to the MTT assay, and this fungus-derived PTX demonstrated robust action against them. Raj et al. [152] demonstrated that Cladosporium oxysporum-derived PTX inhibited HCT-15 cell growth at higher concentrations. They hypothesized that this fungus might serve as a different source of PTX.

[153] isolated the PTX (*Fusarium solani*) from *T. celebica.* They observed that PTX-induced induced DNA fragmentation, caspase-10 activation, apoptosis, and mitochondrial membrane potential loss. Suresh et al. [154] treated NCI-H460 cells with fungal-derived PTX from *A. oryzae*. The treatment resulted in alterations in cell structure, with the cells becoming sphere-shaped. Fungal PTX displayed significant in vitro cytotoxic activity, inducing apoptosis. It is important to remember that this research may utilize different techniques, cell lines, and particular doses. However, taken as a whole, they show that PTX can kill different cancer cell lines. Matrix metalloproteinase 9 (MMP9) activity is decreased by PTX treatment, which is identified to play a crucial control over the tumor microenvironment and the development of cancer [155]. Inhibiting MMP9 activity can help to impede cancer progression.

Fungal-derived PTX also prompts the initiation of cytotoxic activity in mice [156]. The effects of PTX on Sprague Dawley rat breast tissue generated from *Botryodiplodia theobromae* were discovered by [157]. They noticed that the levels of antioxidants such as catalase (CAT), glutathione peroxidases (GPx), and superoxide dismutase (SOD) were raised by PTX. PTX also increased the levels of non-enzymatic antioxidants like glutathione (GSH), vitamin C, and vitamin E. Superoxide dismutase (SOD) is a tumor suppressor protein as well as an intracellular enzyme that guards cells from oxidative damage and breaks down superoxide radicals into hydrogen peroxide [158]. By increasing the levels of antioxidants (CAT, SOD, and GPx), fungal PTX helps to block ROS and their cell-induced effects [159]. In Sprague Dawley rats, COX-2 (cyclooxygenase-2) is recognized for stimulating cancer cell growth. PTX considerably declines the COX-2 expression [157]. By reducing COX-2 levels, fungal PTX may hinder cancer cell growth and enhance the cancer cell's susceptibility to undergo programmed cell death. These findings suggest that fungal-derived PTX exhibits various beneficial effects, including inhibition of MMP9 activity, induction of caspase 3-mediated cytotoxicity, modulation of antioxidant levels, and suppression of COX-2 expression. These effects collectively contribute to the potential anticancer properties of fungal PTX.

## Conclusion

This article aims to give an overview of the molecular and pharmacological aspects of PTX's ability to fight cancer. PTX (marketed as Taxol^®^) is a tetracyclic diterpenoid that was initially discovered in the bark of *Taxus brevifolia*, a Pacific yew tree. It is the first taxane to undergo clinical trials and is an active chemotherapy drug against a wide range of cancers, typically resistant to conventional treatments. The US FDA in 1992 approved it for ovarian cancer treatment and advanced and early-stage breast cancer treatments in 1994 and 1999, respectively. PTX is administered as a second-line drug in monotherapy when combination chemotherapy fails to treat breast cancer or the disease recurs within 6 months of adjuvant chemotherapy. Based on published preclinical data, PTX affects various pathways, causing an overall clinical activity that is not solely dependent on its direct cytotoxic effects on cancer cells. As a result, PTX (including its new formulations) may provide unique and rational therapeutic approaches to managing tumor progression in patients.

## Data Availability

Not applicable.

## Abbreviations

ABC: ATP-binding cassette
ATC: Anaplastic thyroid cancer
BBB: Blood–brain barrier
CAT: Catalase
COX-2: Cyclooxygenase-2
DHA: Docosahexaenoic acid
FDA: Food and Drug Administration
GPx: Glutathione peroxidases
HPLC/LC–MS: High-performance liquid chromatography/mass spectrometry
HPLC: High-performance liquid chromatography
HRPC: Hormone-refractory prostate cancer
LC–ESI-MS: Liquid chromatography–electrospray ionization tandem mass spectrometry
LEP-ETU: Liposome-entrapped
MDR: Multidrug resistance
MMP9: Matrix metalloproteinase
NMR: Nuclear magnetic resonance
NSCLC: Non-small-cell lung cancer
P-gp: P-glycoprotein
PTX-TTHA: Paclitaxel-triethylenetetramine hexaacetic acid conjugate
SOD: Superoxide dismutase
SWNT: Single-walled carbon nanotubes
TLC: Thin-layer chromatography
UV: Ultraviolet
